# Analysis of HDAC6 and BAG3-Aggresome Pathways in African Swine Fever Viral Factory Formation

**DOI:** 10.3390/v7041823

**Published:** 2015-04-08

**Authors:** Raquel Muñoz-Moreno, Lucía Barrado-Gil, Inmaculada Galindo, Covadonga Alonso

**Affiliations:** 1 Department of Biotechnology, Instituto Nacional de Investigación y Tecnología Agraria y Alimentaria (INIA), 28040 Madrid, Spain; E-Mails: raquel.munoz@mssm.edu (R.M.-M.); barrado.lucia@inia.es (L.B.-G.); galindo@inia.es (I.G.); 2 Department of Microbiology, Icahn School of Medicine at Mount Sinai, New York, 10029 NY, USA; 3 Global Health and Emerging Pathogens Institute, Icahn School of Medicine at Mount Sinai, New York, 10029 NY, USA

**Keywords:** African swine fever virus, aggresome, HDAC6, BAG3, viral factories

## Abstract

African swine fever virus (ASFV) is a double-stranded DNA virus causing a hemorrhagic fever disease with high mortality rates and severe economic losses in pigs worldwide. ASFV replicates in perinuclear sites called viral factories (VFs) that are morphologically similar to cellular aggresomes. This fact raises the possibility that both VFs and aggresomes may be the same structure. However, little is known about the process involved in the formation of these viral replication platforms. In order to expand our knowledge on the assembly of ASFV replication sites, we have analyzed the involvement of both canonical aggresome pathways in the formation of ASFV VFs: HDAC6 and BAG3. HDAC6 interacts with a component of the dynein motor complex (dynactin/p150^Glued^) and ubiquitinated proteins, transporting them to the microtubule-organizing center (MTOC) and leading to aggresome formation, while BAG3 is mediating the recruitment of non-ubiquitinated proteins through a similar mechanism. Tubacin-mediated HDAC6 inhibition and silencing of BAG3 pathways, individually or simultaneously, did not prevent ASFV VF formation. These findings show that HDAC6 and Bag3 are not required for VFs formation suggesting that aggresomes and VFs are not the same structures. However, alternative unexplored pathways may be involved in the formation of aggresomes.

## 1. Introduction

African Swine Fever Virus (ASFV) is a large icosahedral enveloped virus that causes a highly contagious disease affecting boars, warthogs, bushpigs and domestic pigs, leading to severe economic losses worldwide. ASFV viral morphogenesis takes place exclusively in cytoplasmic viral factories (VFs), in which the late phase of viral replication also occurs [1].

Viral factories (VFs) are single and large perinuclear structures organized at the microtubule-organizing center (MTOC) in which viral replication takes place and newly synthesized virions are assembled. As we previously reported, incoming viruses are transported to VFs through the interaction between the ASFV structural protein p54 and the motor protein dynein [2]. However, besides this information, little is known about the process involved in the formation of these structures.

There is an increasing interest in the biogenesis of viral replicative structures. VFs morphology resembles aggresomes [3], hence it could be possible that VFs are formed as aggregates of misfolded proteins. Aggresomes are structures formed at the MTOC in response to the aggregation of misfolded proteins [4]. Redirection and isolation of protein aggregates into aggresomes reduces toxicity and facilitates their degradation either by the proteasome or lysosomes after sequestration by autophagy [5].

There are many similarities shared between aggresomes and ASFV VFs. A collapsed cage made of intermediate filament protein vimentin during aggresome formation surrounds protein aggregates. Intriguingly, a vimentin cage at the MTOC also surrounds ASFV VFs, providing a physical scaffold to the factory or acting as a cage to prevent movement of viral components into the cytoplasm.

Aggresomes also recruit a variety of different cellular components to facilitate protein folding as chaperones and mitochondria. ASFV itself encodes a chaperone that is responsible for folding viral protein p72, the major component of the viral icosahedric capsid [6]. ASFV also recruits mitochondria and cellular chaperones such as hsp70 to VFs to facilitate the folding of viral structural proteins [3,7,8].

Core particles of many viruses are also similar in size to protein aggregates (60–100 nm diameter). Therefore, it is conceivable that ASFV viral cores may be recognized as protein aggregates and then delivered to the MTOC [9]. 

These similarities found between factories and aggresomes raised the possibility that ASFV may use the aggresome pathway to facilitate assembly and formation of the VFs.

To date, there are two main pathways described for aggresome formation: the first one involves histone deacetylase 6 (HDAC6) which specifically interacts with dynactin/p150^Glued^, bridging the ubiquitinated proteins to the dynein motors and promoting transport of the cargo towards the microtubule organizing center (MTOC) to enable aggresome formation [10,11]. The second pathway involves Bcl2-associated athanogene 3 protein (BAG3), which acts in a similar fashion but in this case through the binding of non-ubiquitinated cargo.

Since there are ubiquitinated proteins in VFs and some of them are of viral origin [12], we investigated the role of these pathways in the formation of ASFV VFs. 

## 2. Material and Methods

### 2.1. Cells, Viruses and Inhibitors

Vero cells were obtained from the American Type Culture Collection (ATCC, Richmond, VA, USA) and maintained in Dulbecco modified Eagle’s medium (DMEM) supplemented with 5% fetal bovine serum (FBS), 100 U/mL penicillin, 100 μg/mL streptomycin and 2 mM l-glutamine at 37 °C and 5% CO_2_.

The tissue culture-adapted ASFV isolate Ba71V was used in all experiments at a moi of 1 or 5 pfu/cell as indicated [13]. Preparation of viral stocks, titrations and infection procedures were carried out in Vero cells as previously described [13].

Vero cells or silenced BAG3 cells were pretreated with 5 mM MG132 to induce aggresome formation and collected at 16 h. For HDAC6 inhibition, cells were treated with 2 µM tubacin for 3 h previous to infection and then collected at 16 hpi. Simultaneous inhibition of both pathways was performed in shBAG3 cell lines pretreated with 2 µM tubacin for 3 h. Inhibitors were maintained throughout the experiment.

### 2.2. Generation of Stable Cell Lines

To generate stable cell lines expressing BAG3 or Scr control, the commercially available lentiviral expression vector pLVX-Puro (Clontech, Mountain View, CA, USA) was used to clone the proteins of interest. 293T cells were transfected at 80% confluency using Lipofectamine 2000 (Life technologies, Carlsbad, CA, USA) with Opti-MEM (Life technologies) in 10-cm^2^ plates. Plates were previously pretreated with poly-l-lysine (Sigma-Aldrich, St Louis, MI, USA) at a final concentration of 0.1 mg/mL to avoid cell detachment. Co-transfection of pLVX-puro expression vector together with vesicular stomatitis virus glycoprotein (VSV-G) and the human immunodeficiency virus (HIV) gag-pol expressing plasmids was performed to produce pseudotyped lentiviral vectors. 

Supernatants containing the pseudotyped lentiviruses were collected twice at 48 and 72 h postransfection. Cell debris were removed by brief centrifugation at 1000 rpm for 5 min and cleared supernatants were 0.2 μM-filtered and stored at −80 °C until use.

Vero cells at 40% confluence were transduced with the pseudotyped lentiviruses expressing the gene of interest and supplemented with 1 μg/mL of polybrene (EMD Millipore, Billerica, MA, USA). Twenty four hours later, transduced-cells were selected by adding 8 μg/mL of puromycin (Life Technologies). Optimal puromycin working concentration was previously titered in non-transduced cells. Finally, protein expression levels of Vero stable-cell lines were determined by Western Blot (WB).

### 2.3. Immunofluorescence

Cells were seeded and grown on glass coverslips. Mock-infected and infected cells were fixed with 4% paraformaldehyde in PBS for 15 min at room temperature. Following cell fixation, cells were permeabilized with PBS-0.1% Triton X-100 for 10 min at room temperature.

After blocking with bovine serum albumin (BSA; Sigma Aldrich) or normal goat serum (Sigma) for 1 h, cells were stained with primary and secondary antibodies and then incubated with Topro-3 (Life technologies, Carlsbad, CA, USA) 1:1000 in PBS for DNA staining. After washing, coverslips were finally mounted on glass plates and cells were observed under a Leica TCS SPE confocal microscope (Leica-Microsystems, Wetzlar, Germany) using a 63X immersion oil objective. Images were captured with Leica Application Suite advanced fluorescence software (LAS AF).

The primary antibodies used for immunofluorescence assays included the following: anti-ASFV mouse monoclonal antibody p72 (Ingenasa, Madrid, Spain) 1:1000, anti-HDAC6 rabbit polyclonal antibody 1:100 (Santa Cruz, Santa Cruz, CA, USA), anti-tubulin mouse monoclonal antibody 1:2000 (Sigma Aldrich), anti-acetylated tubulin mouse monoclonal 1:1000 (Sigma Aldrich), anti-BAG3 rabbit polyclonal antibody 1:50 (Proteintech, Manchester, UK).

Secondary antibodies were purchased from Molecular Probes and diluted 1:200. Specificity of labeling and absence of signal crossover were determined by examination of single labeled control samples.

### 2.4. Western Blotting

Cells were harvested in Laemmli sample buffer (Life technologies), heated for 5 min at 95 °C and resolved by SDS-PAGE in 12% polyacrylamide-bisacrylamide gels. Afterwards, separated proteins were transferred to a nitrocellulose membrane (Bio-Rad, Hercules, CA, USA) and the non-specific antibody binding sites were blocked with skimmed milk diluted in PBS and then incubated with the specific primary and HRP (Horseradish peroxidase)-conjugated secondary antibodies. Antibodies used for western blotting included: anti-rabbit polyclonal against BAG3 1:500 (Proteintech). As a loading control an anti-mouse antibody against β-tubulin (Sigma) 1:2000 was used. As secondary antibodies, anti-mouse IgG (GE Healthcare, Piscataway, NJ, USA) or anti-rabbit IgG (Bio-Rad) conjugated to horseradish peroxidase were used at a 1:5000 dilution. Precision Protein StrepTactin-HRP Conjugate (Bio-Rad) was used to reveal the ladder Precision Plus Protein WesternC (Bio-Rad). Finally, bands obtained after development with ECL reagent (GE Healthcare, Piscataway, NJ, USA) were detected using a Chemidoc XRSplus Imaging System (Bio-Rad). Band densitometry was performed with Image Lab software (Bio-Rad Inc: 2011) and data were normalized to loading control values.

### 2.5. Statistical Analysis

Bonferroni’s multiple-comparison test was used to compare different experimental groups. GraphPad Prism 5 software and GraphPad Instat 3 software (GraphPad Software, Inc., La Jolla, CA, USA) were used for the statistical analysis. Values were expressed in graph bars as mean ± SD of at least three independent experiments unless otherwise noted. Metrics were normalized to control values and represented in graphics. Asterisks denote statistically significant differences (*** *p* < 0.001, ** *p* < 0.01 and * *p* < 0.05).

## 3. Results and Discussion

**Figure 1:**
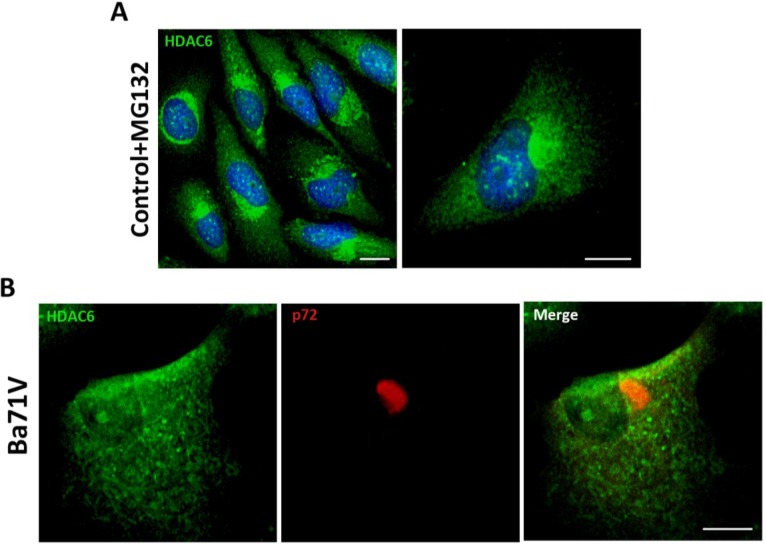
Analysis of histone deacetylase 6 (HDAC6) colocalization with African swine fever virus (ASFV) viral factories (VFs). (**A**) Analysis of HDAC6 expression in Vero cells treated with proteasome inhibitor MG132 (5 mM) for 16 h; (**B**) Cellular localization of p72 viral protein (red) and HDAC6 (green) in ASFV-infected Vero cells (moi = 5 pfu/cell) at 16 hpi. Bar = 10 μm.

First, we induced the formation of aggresome structures by treatment of Vero cells with proteasome inhibitor MG132 (5 mM). After 16 h, aggresomes were visualized by immunofluorescence (IF) using an antibody against HDAC6. As shown in Figure 1A, upon proteasome inhibition, HDAC6 was accumulated in a perinuclear area forming the aggresome. ASFV infection of Vero cells (Ba71V isolate, moi = 5 pfu/cell) resulted in the formation of the characteristic VF, which stained against structural protein p72 as it accumulates in VFs at 16 hpi. However, VFs lacked HDAC6 staining. The absence of colocalization between both proteins, suggested that ASFV VFs were not true aggresomes (Figure 1B). These findings are complementary to previously described results showing that inhibition of HDAC6 with tubacin did not affect ASFV infection. The size, shape or number of VFs found in the cell were not affected by tubacin treatment [14].

The second pathway involved in aggresome formation is mediated by BAG3. Given that many misfolded proteins in aggresomes are not ubiquitinated [15], selective loading of cargo onto dynein should also occur independently of ubiquitin and it is specifically mediated by BAG3. BAG3 directly associates with the microtubule motor dynein through its PxxP domain and mediates a selective transport of misfolded proteins to the aggresome [16]. Therefore, we wanted to analyze the possible role of BAG3 in VF formation as we previously did for HDAC6.

**Figure 2:**
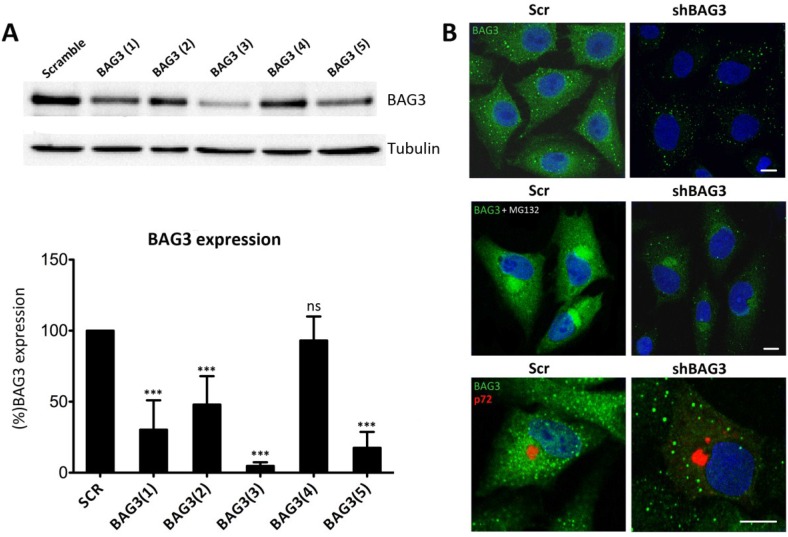
Analysis of Bag3 in VF formation (**A**) Levels of BAG3 silencing among five clones obtained from shBAG3 transduction in Vero cells. BAG3 clone number 3 (BAG3(3)) was the selected candidate for further experiments. Graphics in the lower panel show mean ± SD of WB quantification. (**B**) BAG3 expression in shBAG3(3) Vero cells and control (Scr) (upper panel); with proteasome inhibitor MG132 treatment (5 mM) for 16 h (middle panel) or infected with ASFV (moi = 1 pfu/cell). Capsid viral protein p72 (red) was used to detect VFs (lower panel). Asterisks denote statistically significant differences (*** = *p* < 0.001; ns = non significant). Bar = 10 μm.

Given that there are not specific BAG3 inhibitors available, we silenced BAG3 in Vero cells using shRNA lentiviral transduction methods. A total of five different clones for shBAG3 were tested and WB analysis was performed to screen the stable cell line with the highest silencing level. As seen in Figure 2A, the stable cell line that showed the highest BAG3 knockdown was clone number 3, BAG3(3). It thus became our selected candidate to perform further experiments while the others were discarded. In parallel, IF of shBAG3 Vero cells was performed to ensure that BAG3 levels were reduced when compared to scrambled (Scr) control (Figure 2B).

To further confirm shBAG3 Vero cells were properly silenced, they were pretreated overnight with proteasome inhibitor MG132 (5 mM). As shown in Figure 2B, control cells displayed perinuclear aggresomes by IF when stained with a specific antibody against BAG3. In contrast, treated shBAG3 cells either did not exhibit aggresome formation or they were smaller in size under the same antibody conditions. Once Vero shBAG3 cells were well characterized, we analyzed the effect of BAG3 knockdown in VF formation. To that end, we infected Vero shBAG3 cells (moi = 1 pfu/cell) and then immunostained against BAG3 and viral protein p72. As seen in Figure 2B, confocal microscopy revealed that VF was still formed in Vero shBAG3 cells and BAG3 did not colocalize with p72. The same disposition of VF was found in Vero Scr control cells.

**Figure 3:**
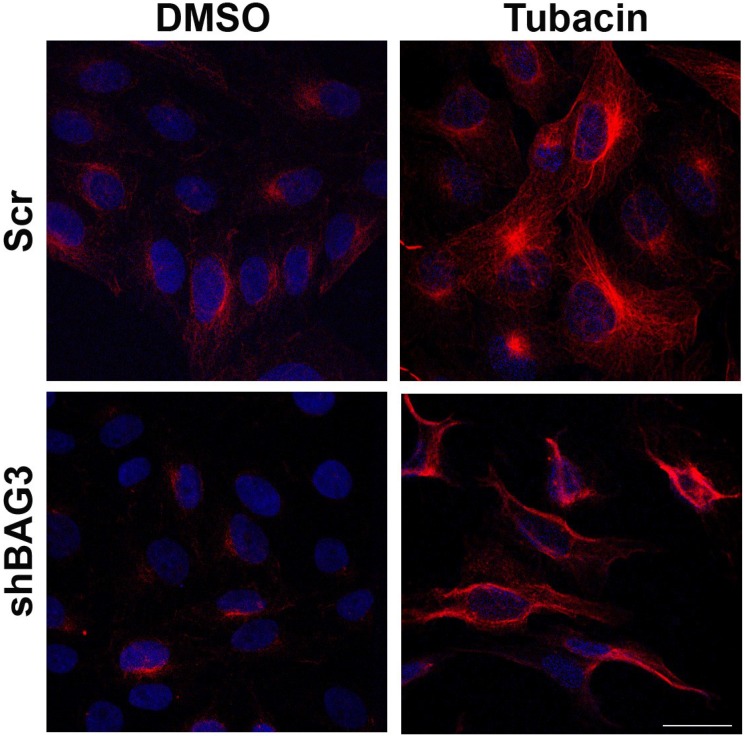
Effect of HDAC6 inhibitor tubacin in shBAG3 cells and Scr control cells. Acetylated tubulin (red) expression in shBAG3 and Scr cells treated with DMSO or HDAC6 inhibitor tubacin (2 µM) for 16 h. Tubacin treated cells showed higher acetylated tubulin staining compared to controls. Bar = 25µm.

After inhibiting separately both aggresome canonical pathways and once confirmed that inhibition of each pathway did not modify ASFV VF formation, our next approach was focused on inhibiting both pathways simultaneously. To that end, shBAG3 Vero cells were pretreated for 3 hours with HDAC6 inhibitor tubacin (2 µM) and subsequently infected with ASFV (moi = 3 pfu/cell). The tubacin solvent DMSO was used as a control. Since HDAC6 is a histone deacetylase, increasing levels of acetylated tubulin can be used to monitor the effect of HDAC6 inhibitor tubacin. As shown in Figure 3, an enhanced expression of acetylated tubulin was found in both shBAG3 stable cell line and in control cells (Scr) pretreated with tubacin.

**Figure 4:**
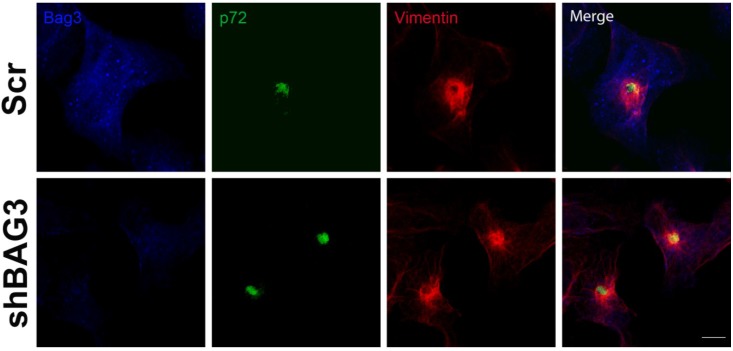
Effect of the inhibition of both BAG3 and HDAC6 pathways in VF formation. Confocal microscopy images of shBAG3 cells treated with tubacin and infected with ASFV (moi = 3 pfu/cell) for 16 h. Viral factories were formed under the inhibition of both pathways. Their morphology was unchanged and the VFs showed the characteristic vimentin cage. Bar = 10 µm.

After 16 h of infection, we could observe the VF (p72) surrounded by the characteristic vimentin cage in our shBAG3 cell line pretreated with tubacin (Figure 4). Hence, inhibition of both BAG3 and HDAC6 pathways still did not affect VF formation.

Based in the morphological features shared between viral factories (VFs) and aggresomes, we have analyzed VF formation as a possible sequestration of cytoplasmic proteins into structures resembling aggresomes. We have tested the two canonical aggresome formation pathways that involve HDAC6 and BAG3 proteins to find out that ASFV VFs did not label with HDAC6 or BAG3 as expected for aggresomes. Also, inhibition of both pathways, either one by one or simultaneously did not alter ASFV VF formation. Hence, although the morphology of ASFV VF is similar to that of aggresomes, the mechanism by which these structures are formed is apparently not related. However, this cannot exclude the possibility of finding new aggresome formation routes that remain unexplored to date. 

## References

[1] Breese S.S., DeBoer C.J. (1966). Electron microscope observations of African swine fever virus in tissue culture cells. Virology.

[2] Alonso C., Miskin J., Hernaez B., Fernandez-Zapatero P., Soto L., Canto C., Rodriguez-Crespo I., Dixon L., Escribano J.M. (2001). African swine fever virus protein p54 interacts with the microtubular motor complex through direct binding to light-chain dynein. J. Virol..

[3] Heath C.M., Windsor M., Wileman T. (2001). Aggresomes resemble sites specialized for virus assembly. J. Cell Biol..

[4] Johnston J.A., Ward C.L., Kopito R.R. (1998). Aggresomes: A cellular response to misfolded proteins. J. Cell Biol..

[5] Garcia-Mata R., Gao Y.S., Sztul E. (2002). Hassles with taking out the garbage: Aggravating aggresomes. Traffic.

[6] Cobbold C., Windsor M., Wileman T. (2001). A virally encoded chaperone specialized for folding of the major capsid protein of African swine fever virus. J. Virol..

[7] Castello A., Quintas A., Sanchez E.G., Sabina P., Nogal M., Carrasco L., Revilla Y. (2009). Regulation of host translational machinery by African swine fever virus. PLoS Pathog..

[8] Rojo G., Chamorro M., Salas M.L., Vinuela E., Cuezva J.M., Salas J. (1998). Migration of mitochondria to viral assembly sites in African swine fever virus-infected cells. J. Virol..

[9] Wileman T. (2007). Aggresomes and pericentriolar sites of virus assembly: Cellular defense or viral design?. Annu. Rev. Microbiol..

[10] Dompierre J.P., Godin J.D., Charrin B.C., Cordelieres F.P., King S.J., Humbert S., Saudou F. (2007). Histone deacetylase 6 inhibition compensates for the transport deficit in Huntington’s disease by increasing tubulin acetylation. J. Neurosci..

[11] Kawaguchi Y., Kovacs J.J., McLaurin A., Vance J.M., Ito A., Yao T.P. (2003). The deacetylase HDAC6 regulates aggresome formation and cell viability in response to misfolded protein stress. Cell.

[12] Hingamp P.M., Leyland M.L., Webb J., Twigger S., Mayer R.J., Dixon L.K. (1995). Characterization of a ubiquitinated protein which is externally located in African swine fever virions. J. Virol..

[13] Enjuanes L., Carrascosa A.L., Moreno M.A., Vinuela E. (1976). Titration of African swine fever (ASF) virus. J. Gen. Virol..

[14] Alonso C., Galindo I., Cuesta-Geijo M.A., Cabezas M., Hernaez B., Munoz-Moreno R. (2013). African swine fever virus-cell interactions: from virus entry to cell survival. Virus Res..

[15] Garcia-Mata R., Bebok Z., Sorscher E.J., Sztul E.S. (1999). Characterization and dynamics of aggresome formation by a cytosolic GFP-chimera. J. Cell Biol..

[16] Gamerdinger M., Kaya A.M., Wolfrum U., Clement A.M., Behl C. (2011). BAG3 mediates chaperone-based aggresome-targeting and selective autophagy of misfolded proteins. EMBO Rep..

